# CORE: A Global Aggregation Service for Open Access Papers

**DOI:** 10.1038/s41597-023-02208-w

**Published:** 2023-06-07

**Authors:** Petr Knoth, Drahomira Herrmannova, Matteo Cancellieri, Lucas Anastasiou, Nancy Pontika, Samuel Pearce, Bikash Gyawali, David Pride

**Affiliations:** 1 Knowledge Media Institute, The Open University Walton Hall, Milton Keynes, UK; 2 Present Address: Oak Ridge National Laboratory Oak Ridge, Oak Ridge, TN USA

**Keywords:** Publishing, Research data

## Abstract

This paper introduces CORE, a widely used scholarly service, which provides access to the world’s largest collection of open access research publications, acquired from a global network of repositories and journals. CORE was created with the goal of enabling text and data mining of scientific literature and thus supporting scientific discovery, but it is now used in a wide range of use cases within higher education, industry, not-for-profit organisations, as well as by the general public. Through the provided services, CORE powers innovative use cases, such as plagiarism detection, in market-leading third-party organisations. CORE has played a pivotal role in the global move towards universal open access by making scientific knowledge more easily and freely discoverable. In this paper, we describe CORE’s continuously growing dataset and the motivation behind its creation, present the challenges associated with systematically gathering research papers from thousands of data providers worldwide at scale, and introduce the novel solutions that were developed to overcome these challenges. The paper then provides an in-depth discussion of the services and tools built on top of the aggregated data and finally examines several use cases that have leveraged the CORE dataset and services.

## Introduction

Scientific literature contains some of the most important information we have assembled as a species, such as how to treat diseases, solve difficult engineering problems, and answer many of the world’s challenges we are facing today. The entire body of scientific literature is growing at an enormous rate with an annual increase of more than 5 million articles (almost 7.2 million papers were published in 2022 according to Crossref, the largest Digital Object Identifier (DOI) registration agency). Furthermore, it was estimated that the amount of research published each year increases by about 10% annually^1^. At the same time, an ever growing amount of research literature, which has been estimated to be well over 1 million publications per year in 2015^2^, is being published as open access (OA), and can therefore be read and processed with limited or no copyright restrictions. As reading this knowledge is now beyond the capacities of any human being, text mining offers the potential to not only improve the way we access and analyse this knowledge^3^, but can also lead to new scientific insights^4^.

However, systematically gathering scientific literature to enable automated methods to process it at scale is a significant problem. Scientific literature is spread across thousands of publishers, repositories, journals, and databases, which often lack common data exchange protocols and other support for inter-operability. Even when protocols are in place, the lack of infrastructure for collecting and processing this data, as well as restrictive copyrights and the fact that OA is not yet the default publishing route in most parts of the world further complicate the machine processing of scientific knowledge.

To alleviate these issues and support text and data mining of scientific literature we have developed CORE (https://core.ac.uk/). CORE aggregates open access research papers from thousands of data providers from all over the world including institutional and subject repositories, open access and hybrid journals. CORE is the largest collection of OA literature–at the time of writing this article, it provides a single point of access to scientific literature collected from over ten thousand data providers worldwide and it is constantly growing. It provides a number of ways for accessing its data for both users and machines, including a free API and a complete dump of its data.

As of January 2023, there are 4,700 registered API users and 2,880 registered dataset and more than 70 institutions have registered to use CORE Recommender in their repository systems.

The main contributions of this work are the development of CORE’s continuously growing dataset and the tools and services built on top of this corpus. In this paper, we describe the motivation behind the dataset’s creation and the challenges and methods of assembling it and keeping it continuously up-to-date. Overcoming the challenges posed by creating a collection of research papers of this scale required devising innovative solutions to harvesting and resource management. Our key innovations in this area which have contributed to the improvement of the process of aggregating research literature include:

1. Devising methods to extend the functionality of existing widely-adopted metadata exchange protocols which were not designed for content harvesting, to enable efficient harvesting of research papers’ full texts.
2. Developing a novel harvesting approach (referred to here as CHARS) which allows us to continuously utilise the available compute resources while providing improved horizontal scalability, recoverability, and reliability.
3. Designing an efficient algorithm for scheduling updates of harvested resources which optimises the recency of our data while effectively utilising the compute resources available to us.

This paper is organised as follows. First, in the remainder of this section, we present several use cases requiring large scale text and data mining of scientific literature, and explain the challenges in obtaining data for these tasks. Next, we present the data offered by CORE and our approach for systematically gathering full text open access articles from thousands of repositories and key scientific publishers.

### Terminology

In digital libraries the term **record** is typically used to denote a digital object such as text, image, or video. In this paper and when referring to data in CORE, we use the term **metadata record** to refer to the metadata of a research publication, i.e. the title, authors, abstract, project funding details, etc., and the term **full text record** to describe a metadata record which has an associated full text.

We use the term **data provider** to refer to any database or a dataset from which we harvest records. Data providers harvested by CORE include disciplinary and institutional repositories, publishers and other databases.

When talking about **open access** (OA) to scientific literature, we refer to the Budapest Open Access Initiative (BOAI) definition which defines OA as “free availability on the public internet, permitting any users to read, download, copy, distribute, print, search, or link to the full texts of these articles, crawl them for indexing, pass them as data to software, or use them for any other lawful purpose” (https://www.budapestopenaccessinitiative.org/read). There are two routes to open access, 1) OA repositories and 2) OA journals. The first can be achieved by self-archiving (depositing) publications in repositories (green OA), and the latter by directly publishing articles in OA journals (gold OA).

### Text and Data Mining of Scientific Literature

Text and data mining (TDM) is the discovery by a computer of new, previously unknown information, by automatically extracting information from different written resources (http://bit.ly/jisc-textm). The broad goal of TDM of scientific literature is to build tools that can retrieve useful information from digital documents, improve access to these documents, or use these documents to support scientific discovery. OA and TDM of scientific literature have one thing in common–they both aim to improve access to scientific knowledge for people. While OA aims to widen the availability of openly available research, TDM aims to improve our ability to discover, understand and interpret scientific knowledge.

**Fig. 1:**
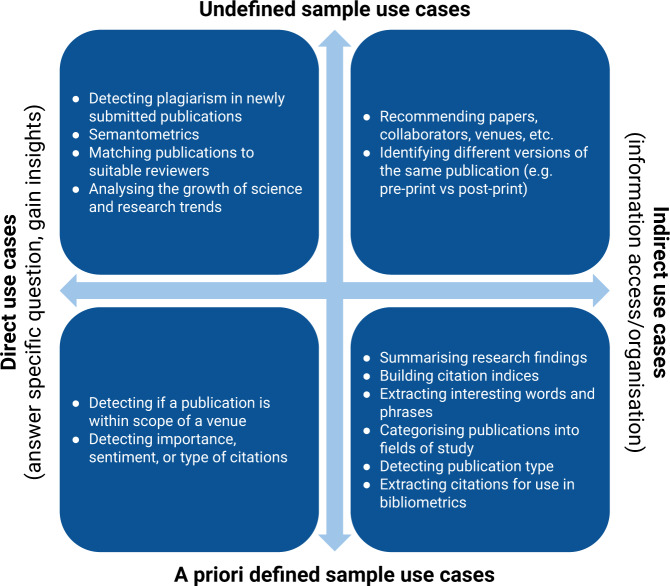
Example uses cases of text and data mining of scientific literature. Depending on data needs, TDM uses can be categorised into a) a priori defined sample use cases, and b) undefined sample use cases. Furthermore, TDM use cases can broadly be categorised into 1) indirect applications which aim to improve access to and organisation of literature and 2) direct applications which focus on answering specific questions or gaining insights.

TDM of scientific literature is being used in a growing number of applications, many of which were until recently not viable due to the difficulties associated with accessing the data from across many publishers and other data providers. Because many use cases involving text and data mining can only realise their full potential when they are executed on an as large corpus of research papers as possible, these data access difficulties have rendered many of the uses cases described below very difficult to achieve. For example, to reliably detect plagiarism in newly submitted publications it is necessary to have access to an always up-to-date dataset of published literature spanning all disciplines. Based on data needs, scientific literature TDM use cases can be broadly categorised into the following two categories, which are shown in Fig. 1:

- **A priori defined sample use cases:** Use cases which require access to a subset of scientific publications that can be specified prior to the execution of the use case. For example, gathering the list of all trialled treatments for a particular disease in the period 2000–2010 is a typical example of such a use case.
- **Undefined sample use cases:** Use cases which cannot be completed using data samples that are defined a priori. The execution of such use cases might require access to data not known prior to the execution or may require access to all data available. Plagiarism detection is a typical example of such use case.

However, there are a number of factors that significantly complicate access to data for these applications. The needed data is often spread across many publishers, repositories, and other databases, often lacking interoperability (these factors will be further discussed in the next section). Consequently, researchers and developers working in these areas typically invest a considerable amount of time in corpus collection, which could be up to 90% of the total investigation time^5^. For many, this task can even prove impossible due to technical restrictions and limitations of publisher platforms, some of which will be discussed in the next section. Consequently, there is a need for a global, continuously updated, and downloadable dataset of full text publications to enable such analysis.

### Challenges in machine access to scientific literature

Probably the largest obstacle to the effective and timely retrieval of relevant research literature is that it may be stored in a wide variety of locations with little to no interoperability: repositories of individual institutions, publisher databases, conference and journal websites, pre-print databases, and other locations, each of which typically offers different means for accessing their data. While repositories often implement a standard protocol for metadata harvesting, the Open Archives Initiative Protocol for Metadata Harvesting (OAI-PMH), publishers typically allow access to their data through custom made APIs, which are not standardised and are subject to changes^6^. Other data sources may provide static data dumps in a variety of formats or not offer programmatic access to their data at all.

**Fig. 2:**
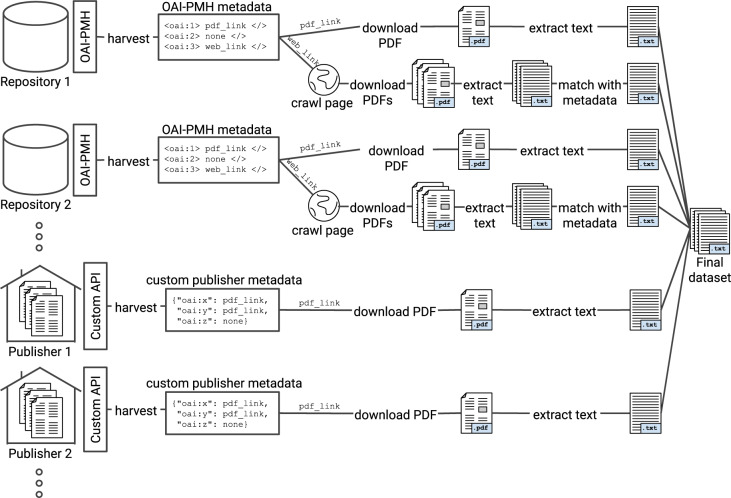
Example illustration of the data collection process. The figure depicts the typical minimum steps which are necessary to produce a dataset for TDM of scientific literature. Depending on the use case, tens or hundreds of different data sources may need to be accessed, each potentially requiring a different process–for example accessing a different set of API methods or a different process for downloading publication full text. Furthermore, depending on the use case, additional steps may be needed, such as extraction of references, identification of duplicate items or detection of the publication’s language. In the context of CORE, we provide the details of this process in Section Methods.

However, even when publication metadata can be obtained, other steps involved in the data collection process complicate the creation of a final dataset suitable for TDM applications. For example, the identification of scientific publications within all downloaded documents, matching these publications correctly to the original publication metadata, and their conversion from formats used in publishing, such as the PDF format, into a textual representation suitable for text and data mining, are just some of the additional difficulties involved in this process. The typical minimum steps involved in this process are illustrated in Fig. 2. As there are no widely adopted solutions providing interoperability across different platforms, custom harvesting solutions need to be created for each.

### Challenges in systematically gathering open access research literature

Open access journals and repositories are increasingly becoming the central providers of open access content, in part thanks to the introduction of funder and institutional open access policies^7^. Open access repositories include institutional repositories such as the University of Cambridge Repository https://www.repository.cam.ac.uk/, and subject repositories such arXiv https://arxiv.org/. As of February 2023, there are 6,015 open access repositories indexed in the Directory of Open Access Repositories http://v2.sherpa.ac.uk/opendoar/ (OpenDOAR), as well as 18,935 open access journals indexed in the Directory of Open Access Journals https://doaj.org/ (DOAJ). However, open access research literature can be stored in a wide variety of other locations, including publisher and conference websites, individual researcher websites, and elsewhere. Consequently, a system for harvesting open access content needs to be able to harvest effectively from thousands of data providers. Furthermore, a large number of open access repositories (69.4% of repositories indexed in OpenDOAR as of January 2018) expose their data through the OAI-PMH protocol while often not providing any alternatives. An open access harvesting system therefore also needs to be able to effectively utilise OAI-PMH for open access content harvesting. However, these two requirements–harvesting from thousands of data providers and utilising OAI-PMH for content harvesting–pose a number of significant scalability challenges.

### Challenges related to harvesting from thousands of data providers

Open access data providers vary greatly in size, with some hosting millions of documents while others host a significantly lower number. New documents are added and old documents are often updated by data providers daily.

Different geographic locations and internet connection speeds may result in vastly differing times needed to harvest information from different providers, even when their size in terms of publication numbers is the same. As illustrated in Table 1, there are also a variety of OAI-PMH implementations across commonly used repository platforms providing significantly different harvesting performance. To construct this table, we analysed OAI-PMH metadata harvesting performances of 1,439 repositories in CORE, covering eight different repository platforms. It should be noted that the OAI-PMH protocol only necessitates metadata to be expressed in the Dublin Core (DC) format. However, it also can be extended to express the metadata in other formats. Because the Dublin-Core standard is constrained to just 15 elements, it is not uncommon for OAI-PMH repositories to also use and extended metadata format such as Rioxx (https://rioxx.net) or the OpenAIRE Guidelines (https://www.openaire.eu/openaire-guidelines-for-literature-institutional-and-thematic-repositories).

**Table 1:** Comparing different repository metadata download speeds per repository platform.

IR Software	No. of repos.	Average	Median	*σ*^2^
DSpace	659	147.29	71.64	1.07*e* + 06
EPrints	402	35.48	29.14	1.98*e* + 03
Digital Commons	149	28.57	11.47	3.70*e* + 04
OPUS	74	39.56	23.84	2.42*e* + 03
OJS	70	11.13	10.13	5.77*e* + 01
dLibra	55	13.39	0.35	2.71*e* + 03
Fedora	16	71.59	32.13	2.18*e* + 04
Invenio	14	136.17	70.42	6.97*e* + 04

The results are for an average speed and the speed of a median repository and are expressed in the number of records transferred per second.

Additionally, harvesting is limited not only by factors related to the data providers, but also by the compute resources (hardware) available to the aggregator. As many use cases listed in the Introduction, such as in plagiarism detection or systematic review automation, require access to very recent data, ensuring that the harvested data stays recent and that the compute resources are utilised efficiently both pose significant challenges.

To overcome these challenges, we designed the CORE Harvesting System (CHARS) which relies on two key principles. The first is the application of the microservices software principles to open access content harvesting^8^. The second is our strategy we denote **pro-active harvesting**, which means that providers are scheduled automatically according to current need. This strategy is implemented in the harvesting Scheduler (Section CHARS_architecture). The Scheduler uses a formula we designed for prioritising data providers.

The combination of the Scheduler with CHARS microservices architecture enables us to schedule harvesting according to current compute resource utilisation, thus greatly increasing our harvesting efficiency. Since switching from a fixed-schedule approach described above to pro-active harvesting, we have been able to greatly improve the data recency of our collection as well as to increase the size of the collection threefold within the span of three years.

### Challenges related to the use of OAI-PMH protocol for content harvesting

As explained above, OAI-PMH is currently the standard method for exchanging data across repositories. While the OAI-PMH protocol was originally been designed for metadata harvesting only, it has been, due to its wide adoption and lack of alternatives, used as an entry point for full text harvesting. Full text harvesting is achieved by extracting URLs from the metadata records collected through OAI-PMH, the extracted URLs are then used to discover the location of the actual resource^9^. However, there are a number of limitations of the OAI-PMH protocol which make it unsuitable for large-scale content harvesting:

1. It directly supports only metadata harvesting, meaning additional functionality has to be implemented in order to use it for content harvesting.
2. The location of full text links in the OAI-PMH metadata is not standardised and the OAI-PMH metadata records typically contain multiple links. From the metadata it is not clear which of these links points to the described representation of the resource and in many cases none of them does so directly. Therefore, all possible links to the resource itself have to be extracted from the metadata and tested to identify the correct resource. Furthermore, OAI-PMH does not facilitate any validation for ensuring the discovered resource is truly the described resource. In order to overcome this issues, the adoption of the RIOXX https://rioxx.net/ metadata format or the OpenAIRE guidelines https://guidelines.openaire.eu/ has been promoted. However, the issue of unambiguously connecting metadata records and the described resource is still present.
3. The architecture of the OAI-PMH protocol is inherently sequential, which makes it ill-suited for harvesting from very large repositories. This is because the processing of large repositories cannot be parallelised and it is not possible to recover the harvesting in case of failures.
4. Scalability across different implementations of OAI-PMH differs dramatically. Our analysis (Table 1) shows that performance can differ significantly also when only a single repository software is considered^10^.
5. Other limitations include difficulties in incremental harvesting, reliability issues, metadata interoperability issues, and scalability issues^11^.

We have designed solutions to overcome a number of these issues, which have enabled us to efficiently and effectively utilise OAI-PMH to harvest open access content from repositories. We present these solutions in Section Using OAI-PMH for content harvesting. While we currently rely on a variety of solutions and workarounds to enable content harvesting through OAI-PMH, most of the limitations listed in this section could also be addressed by adopting more sophisticated data exchange protocols, such as the ResourceSync (http://www.openarchives.org/rs/1.1/resourcesync) protocol which was designed with content harvesting in mind^10^ and the adoption in the systems of data providers we support.

## Results

### Our solution

In the above sections we have highlighted a critical need for many researchers and organisations globally for large-scale always up-to-date seamless machine access to scientific literature originating from thousands of data providers at full text level. Providing this seamless access has become both a defining goal and a feature of CORE and has enabled other researchers to design and test innovative methods on CORE data, often powered by artificial intelligence processes. In order to put together this vast continuously updated dataset, we had to overcome a number of research challenges, such as those related to the lack of interoperability, scalability, regular content synchronisation, content redundancy and inconsistency. Our key innovation in this area is **the improvement of the process of aggregating research literature**, as specified in the Introduction section.

This underpinning research has allowed CORE to become a leading provider of open access papers. The amount of data made available by CORE has been growing since 2011^12^ and is continuously kept up to date. As of February 2023, CORE provides access to over 291 million metadata records and 32.8 million full text open access articles, making it the world’s largest archive of open access research papers, significantly larger than PubMed, arXiv and JSTOR datasets.

Whilst there are other publication databases that could be initially viewed as similar to CORE, such as BASE or Unpaywall, we will demonstrate the significant differences that set CORE apart and show how it provides access to a unique, harmonised corpus of Open Access literature. A major difference between these existing services is that CORE is completely free to use for the end user, it hosts full text content, and offers several methods for accessing its data for machine processing. Consequently, it removes the need to harvest and pre-process full text for text mining, since CORE provides plain text access to the full texts via its raw data services, eliminating the need for text and data miners to work on PDF formats. A detailed comparison of other publication databases is provided in the Discussion. In addition, CORE enables building powerful services on top of the collected full texts, supporting all the categories of use cases outlined in the Use cases section.

As of today, CORE provides three services for accessing its raw data: API, dataset, and a FastSync service. The CORE API provides real-time machine access to both metadata and full texts of research papers. It is intended for building applications that need reliable access to a fraction of CORE data at any time. CORE provides a RESTful API. Users can register for an API key to access the service. Full documentation and Python notebooks containing code examples can be found on the CORE documentation pages online (https://api.core.ac.uk/docs/v3). The CORE Dataset can be used to download CORE data in bulk. Finally, CORE FastSync enables third party systems to keep an always up to date copy of all CORE data within their infrastructure. Content can be transferred as soon as it becomes available in CORE using a data synchronisation service on top of the ResourceSync protocol^13^ optimised by us for improved synchronisation scalability with an on-demand resource dumps capability. CORE FastSync provides fast, incremental and enterprise data synchronisation.

CORE is the largest up-to-date full text open access dataset as well as one of the most widely used services worldwide supporting access to freely available research literature. CORE regularly releases data dumps licensed as ODC-By, making the data freely available for both commercial and non-commercial purposes. Access to CORE data via the API is provided freely to individuals conducting work in their own personal capacity and to public research organisations for unfunded research purposes. CORE offers licenses to commercial organisations wanting to use CORE services to obtain a convenient way of accessing CORE data with a guaranteed level of service support. CORE is operated as a not-for-profit entity by The Open University and this business model makes it possible for CORE to remain free for the >99.99% of its users.

A large number of commercial organisations have benefited from these licenses in areas as diverse as plagiarism detection in research, building specialised scholarly publication search engines, developing scientific assistants and machine translation systems and supporting education etc. https://core.ac.uk/about/endorsements/partner-projects. The CORE data services–CORE API and Dataset, have been used by over 7,000 experts to analyse data, develop text-mining applications and to embed CORE into existing production systems.

Additionally, more than 70 repository systems have registered to use the CORE Recommender and the service is notably used by prestigious institutions, including the University of Cambridge and by popular pre-prints services such as arXiv.org. Other CORE services are the CORE Discovery and the CORE Repository Dashboard. The first was released on July 2019 and at the time of writing it has more than 5000 users. The latter is a tool designed specifically for repository managers which provides access to a range of tools for managing the content within their repositories. The CORE Repository Dashboard is currently used by 499 users from 36 countries.

In the rest of this paper we describe the CORE dataset and the methods of assembling it and keeping it continuously up-to-date. We also present the services and tools built on top of the aggregated corpus and provide several examples of how the CORE dataset has been used to create real-world applications addressing specific use-cases.

As highlighted in the Introduction, CORE is a continuously growing dataset of scientific publications for both human and machine processing. As we will show in this section, it is a global dataset spanning all disciplines and containing publications aggregated from more than ten thousand data providers including disciplinary and institutional repositories, publishers, and other databases. To improve access to the collected publications, CORE performs a number of data enrichment steps. These include metadata and full text extraction, language and DOI detection, and linking with other databases. Furthermore, CORE provides a number of services which are built on top of the data: a publications recommender (https://core.ac.uk/services/recommender/), CORE Discovery service (https://core.ac.uk/services/discovery/) (a tool for discovering OA versions of scientific publications), and a dashboard for repository managers (https://core.ac.uk/services/repository-dashboard/).

### Dataset size

As of February 2023, CORE is the world’s largest dataset of open access papers (comparison with other systems is provided in the Discussion). CORE hosts over 291 million metadata records including over 34 million articles with full text written in 82 languages and aggregated from over ten thousand data providers located in 150 countries. Full details of CORE Dataset size are presented in Table 2. In the table, “Metadata records” represent all valid (not retracted, deleted, or for some other reason withdrawn) records in CORE. It can be seen that about 13% of records in CORE contain full text. This number represents records for which a manuscript was successfully downloaded and converted to plain text. However, a much higher proportion of records contains links to additional freely available full text articles hosted by third-party providers. Based on analysing a subset of our data, we estimate that about 48% of metadata records in CORE fall into this category, indicating that CORE is likely to contain links to open access full texts for 139 million articles. Due to the nature of academic publishing there will be instances where multiple versions of the same paper are deposited in different repositories. For example, an early version of an article can be deposited by an author to a pre-print server such as arXiv or BiorXiv and then a later version uploaded to an institutional repository. Identifying and matching these different versions is a significant undertaking. CORE has carried out research to develop techniques based on locality sensitive hashing for duplicates identification^8^ and integrated these into its ingestion pipeline to link versions of papers from across the network of OA repositories and group these under a single *works* entity. The large number of records in CORE translates directly into the size of the dataset in bytes as the uncompressed version of the dataset including PDFs is about 100 TB. The compressed version of the CORE dataset with plain texts only amounts to 393 GB and uncompressed to 3.5 TBs.

**Table 2:** Size of the CORE collection as of January 2023.

Metadata records	291,151,257
Records with full text	32,812,252
Records with abstract	94,521,867
Records with full-text link	139,000,000^†^
Data providers	10,744
Number of CORE data provider countries	150
Estimated number of languages of collected content	118

^†^Estimate based on analysis.

Recent studies have estimated that around 24%–28% of all articles are available **free to read**^2,14^. There are a number of reasons why the proportion of full text content in CORE is lower than these estimates. The main reason is likely that a significant proportion of the free to read articles represents content hosted on platform with many restrictions for machine accessibility, i.e. some repositories severely restrict or fully prohibit content harvesting^9^.

**Fig. 3:**
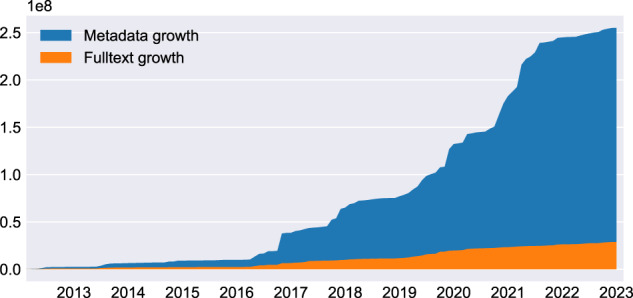
Growth of records in CORE per month since February 2012. “Full text growth” represents growth of records containing full text, while “Metadata growth” represents growth of records without full text, i.e. the two numbers do not overlap. The two area plots are stacked on top of each other, their sum therefore represents the total number of records in CORE.

**Fig. 4:**
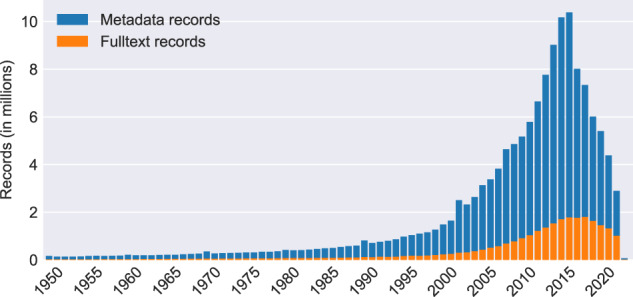
Age of publications in CORE. Similarly as in Fig. 3, the “Metadata” and “Full text” records bars are stacked on top of each other.

The growth of CORE has been made possible thanks to the introduction of a novel harvesting system and the creation of an efficient harvesting scheduler, both of which are described in the Methods section. The growth of metadata and full text records in CORE is shown in Fig. 3. Finally, Fig. 4 shows age of publications in CORE.

### Data sources and languages

As of February 2023, CORE was aggregating content from 10,744 data sources. These data sources include institutional repositories (for example the USC Digital Library or the University of Michigan Library Repository), academic publishers (Elsevier, Springer), open access journals (PLOS), subject repositories, including those hosting eprints (arXiv, bioRxiv, ZENODO, PubMed Central) and aggregators (e.g. DOAJ). The ten largest data sources in CORE are shown in Table 3. To calculate the total number of data providers in CORE, we consider aggregators and publishers as one data source despite each aggregating data from multiple sources. A full list of all data providers can be found on the CORE website. (https://core.ac.uk/data-providers).

**Table 3:** The ten largest data providers in CORE.

Name	Number of documents
Crossref	109,998,842
USC Digital Library	10,956,842
CiteSeerX	6,540,649
Directory of Open Access Journals	4,496,977
Gallica, Bibliotheque Numerique	3,437,495
University of Michigan Library Repository	3,316,595
PubMed Central	2,572,251
FigShare	2,449,457
NARCIS	2,288,374
Elsevier - Publisher Connector	1,682,186

Content aggregators such as DOAJ, Elsevier, and NARCIS are presented as one data source despite collecting data from multiple sources themselves.

**Fig. 5:**
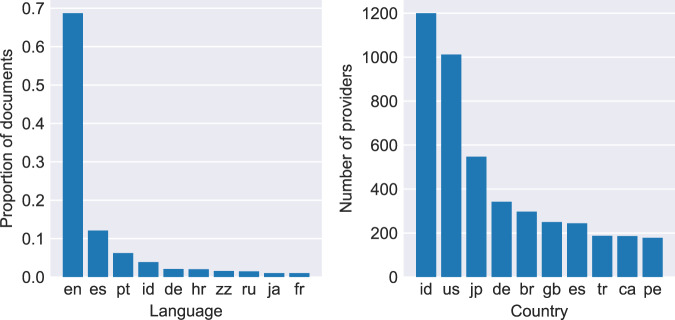
Top ten languages and top ten provider locations in CORE.

The data providers aggregated by CORE are located in 150 different countries. Figure 5 shows the top ten countries in terms of number of data providers aggregated by CORE from each country alongside the top ten languages. The geographic spread of repositories is largely reflective of the size of the research economy in those countries. We see the US, Japan, Germany, Brazil and the UK all in the top six. One result that at first may appear surprising is the significant number of repositories in Indonesia, enough to place them at the top of the list. An article in Nature in 2019 showed that Indonesia may be the world’s OA leader, finding that 81% of 20,000 journal articles published in 2017 with an Indonesia-affiliated author are available to read for free somewhere online. (https://www.nature.com/articles/d41586-019-01536-5). Additionally, there are a large number of Indonesian open-access journals registered with Crossref. This subsequently leads to a much higher number of individual repositories in this country.

As part of the enrichment process, CORE performs language detection. Language is either extracted from the attached metadata where available or identified automatically from full text in case it is not available in metadata. More than 80% of all documents with language information are in English. Overall, CORE contains publications in a variety of languages, the top 10 of which are shown in Fig. 5.

### Document types

**Fig. 6:**
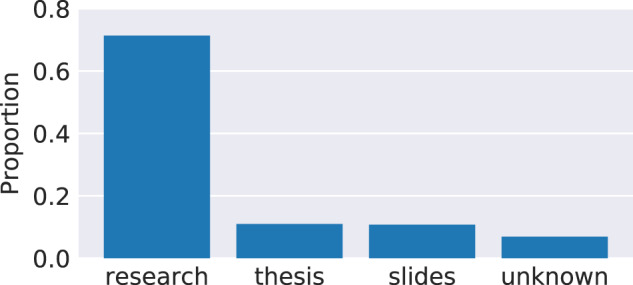
Distribution of document types.

The CORE dataset comprises a collection of documents gathered from various sources, many of which contain articles of different types. Consequently, aside of research articles from journals and conferences, it includes other types of research outputs such as research theses, presentations, and technical reports. To distinguish different types of articles, CORE has implemented a method of automatically classifying documents into one of the following four categories^15^: (1) research article, (2) thesis, (3) presentation, (4) unknown (for articles not belonging into any of the previous three categories). This method is based on a supervised machine learning model trained on article full texts. Figure 6 shows the distribution of articles in CORE into these four categories. It can be seen that the collection aggregated by CORE consists predominantly of research articles. We have observed in the data collected from repositories that the vast majority of research theses deposited in repositories has full text associated with the metadata. As this is not always the case for research articles, and as Fig. 6 is produced on articles with full text only, we expect that the proportion of research articles compared to research theses in CORE is actually higher across the entire collection.

### Research disciplines

**Fig. 7:**
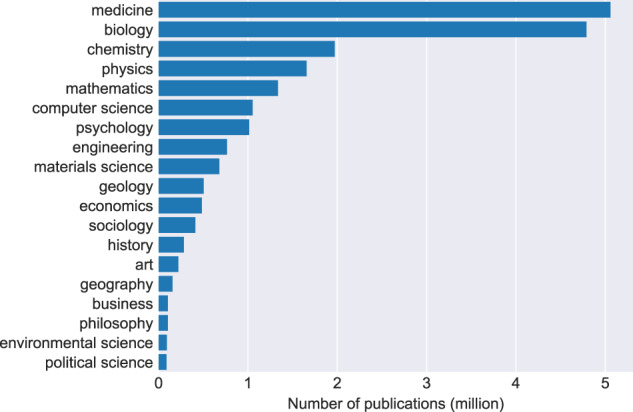
Subject distribution of a sample of 20,758,666 CORE publications.

To analyse the distribution of disciplines in CORE we have leveraged a third-party service. Figure 7 shows a subject distribution of a sample of 20,758,666 publications in CORE. For publications with multiple subjects we count the publication towards each discipline.

The subject for each article was obtained using Microsoft Academic (https://academic.microsoft.com/home) prior to its retirement in November 2021. Our results are consistent with other studies, which have reported Biology, Medicine, and Physics to be the largest disciplines in terms of number of publications^16,17^, suggesting that the distribution of articles in CORE is representative of research publications in general.

### Additional CORE Tools and Services

CORE has built several additional tools for a range of stakeholders including institutions, repository managers and researchers from across all scientific domains. Details of usage of these services is covered in the Uptake of CORE section.

#### Dashboard

The Dashboard provides a suite of tools for repository management, content enrichment, metadata quality assessment and open access compliance checking. Further, it can provide statistics regarding content downloads and suggestions for improving the efficiency of harvesting and the quality of metadata.

#### Discovery

CORE Discovery helps users to discover freely accessible copies of research papers. There are several methods for interacting with the Discovery tool. First, as a plugin for repositories, enriching metadata only pages in repositories with links to open access copies of full text documents. Second, via a browser extension for researchers and anyone interested in reading scientific documents. And finally as an API service for developers.

#### Recommender

The recommender is a plugin for repositories, journal systems and web interfaces that provides suggestions on relevant articles to the one currently displayed. Its purpose is to support users in discovering articles of interest from across the network of open access repositories. It is notably used by prestigious institutions, including the University of Cambridge and by popular pre-prints services such as arXiv.org.

#### OAI Resolver

An OAI (Open Archives Initiative) identifier is a unique identifier of a metadata record. OAI identifiers are used in the context of repositories using the Open Archives Initiative Protocol for Metadata Harvesting (OAI-PMH). OAI Identifiers are viable persistent identifiers for repositories that can be, as opposed to DOIs, minted in a distributed fashion and cost-free, and which can be resolvable directly to the repository rather than to the publisher. The CORE OAI Resolver can resolve any OAI identifier to either a metadata page of the record in CORE or route it directly to the relevant repository page. This approach has the potential to increase the importance of repositories in the process of disseminating knowledge.

### Uptake of CORE

As of February 2023, CORE averages over 40 million monthly active users and is the top 10th website in the category Science and Education according to SimilarWeb (https://www.similarweb.com/). There are currently 4,700 registered API users and 2,880 registered dataset users. The CORE Dashboard is currently used by 499 institutional repositories to manage their open access content, monitor content download statistics, manage issues with metadata within the repository and ensure compliance with OA funder policies, notably REF in the U.K. The CORE Discovery plugin has been integrated into 434 repositories and the browser extension has been downloaded by more than 5,000 users via the Google Chrome Web Store (https://chrome.google.com/webstore/category/extensions). The CORE Recommender has been embedded in 70 repository systems including the University of Cambridge and arXiv.

## Discussion

In this section we discuss differences between CORE and other open access aggregation services and present several real-word use cases where CORE was used to develop services to support science. In this section we also present our future plans.

### Existing open access aggregation services

Currently there are a number of open access aggregation services available (Table 4), with some examples being BASE (https://base-search.net/), OpenAIRE (https://www.openaire.eu/), Unpaywall (http://unpaywall.org/), Paperity (https://paperity.org/). BASE (Bielfield Academic Search Engine) is a global metadata harvesting service. It harvests repositories and journals via OAI-PMH and exposes the harvested content through an API and a dataset. OpenAIRE is a network of open access data providers who support open access policies. Even though in the past the project focused on European repositories, it has recently expanded by including institutional and subject repositories from outside Europe. A key focus of OpenAIRE is to assist the European Council to monitor compliance of its open access policies. OpenAIRE data is exposed via an API. Paperity is a service which harvests publications from open access journals. Paperity harvests both metadata and full text but does not host full texts. SHARE (Shared Access Research Ecosystem) is a harvester of open access content from US repositories. Its aim is to assist with the White House Office of Science and Technology Policy (OSTP) open access policies compliance. Even though SHARE harvests both metadata and full text it does not host the latter. Unpaywall is not primarily a harvester, but rather collects content from Crossref, whenever a free to read available version can be retrieved. It processes both metadata and full text but does not host them. It exposes the discovered links to documents through an API.

**Table 4:** Comparison of OA aggregation services.

	Hosted full text	All metadata records	Records with OA links	API	Dataset	Data sync
**CORE**	32.8 m	291 m	139 m	Y	Y	Y^†^
**BASE**	0	~300 m	~60%	Y	N	N
**OpenAIRE**	19 m	149 m	n/a	N	N	N
**Unpaywall**	0	142.7 m (via Crossref)	46.4 m	Y	Y^⬦^	Y^•^
**Paperity**	0	9.8 m	n/a	N	N	N
**SHARE**	0	57 m	n/a	N	N	N

^*^The full texts hosted by OpenAIRE are not available for download. ^⬦^The dataset provided by Unpaywall contains only links, not full metadata or full text as in the case of CORE. ^†^CORE uses the FastSync mechanism for data sync. ^•^Unpaywall provides data sync as part of its premium service.

CORE differs from these services in a number of ways. CORE is currently the largest database of full text OA documents. In addition, CORE offers via its API a rich metadata record for each item in its collection which includes additional enrichments, contrary, for example, to Unpaywall’s API, which focuses only on delivering to the user information as to whether a free to read version is available. CORE also provides the largest number of links to OA content. To simplify access to data for end users it provides a number of ways for accessing its collection. All of the above services are free to use for research purposes however both CORE and Unpaywall also offer services to commercial partners on a paid-for basis.

### Existing publication databases

Apart from OA aggregation services, a number of other services exists for searching and downloading scientific literature (Table 5). One of the main publication databases is Crossref (https://www.crossref.org/), an authoritative index of DOI identifiers. Its primary function is to maintain metadata information associated with each DOI. The metadata stored by Crossref includes both OA and non-OA records. Crossref does not store publication full text, but for many publications provides full text links. As of February 2023, 5.9 m records in Crossref were associated with an explicit Creative Commons license (we have used the Crossref API to determine this number). Although Crossref provides an API, it does not offer its data for download in bulk, or provide a data sync service.

**Table 5:** Comparison of publication databases.

	Free to use	Hosted full text	API	Dataset	Data sync
**CORE**	Y	Y	Y	Y	Y
**Crossref**	Y	N	Y	N	N
**Scopus**	N	Y	Y	N	N
**Web of Science**	N	Y	Y	N	N
**Google Scholar**	n/a^*^	N	N	N	N
**Semantic Scholar**	Y	Y	Y	Y	N
**Dimensions**	Y	Y	Y	N	N
**1findr**	N	Y	N	N	N

By “free to use” we mean whether the database is free to access using automated methods (e.g. via an API). ^*^Google Scholar does not provide any means for accessing their data programmatically.

The remaining services from Table 5 can be roughly grouped into the following two categories: 1) citation indices, 2) academic search engines and scholarly graphs. The two major citation indices are Elsevier’s Scopus (https://www.elsevier.com/solutions/scopus) and Clarivate’s Web of Science (https://clarivate.com/webofsciencegroup/solutions/web-of-science/), both of which are premium subscription services. Google Scholar, the best known academic search engine does not provide an API for accessing its data and does not permit crawling its website. Semantic Scholar (https://www.semanticscholar.org/) is a relatively new academic search service which aims to create an “intelligent academic search engine”^18^. Dimensions (https://www.dimensions.ai/) is a service focused on data analysis. It integrates publications, grants, policy documents, and metrics. 1findr (https://1findr.1science.com/home) is a curated abstract indexing service. It provides links to full text, but no API or a dataset for download.

### The added value of CORE

There are other services that claim to provide access to a large dataset of open access papers. In particular, Unpaywall^2^, claim to provide access to 46.4 million free to read articles, and BASE, who state they provide access to full texts of about 60% of their 300 million metadata records. However, these statistics are not directly comparable to the numbers we report and are a product of a different focus of these two projects. This is because both the analysis of BASE and now Unpaywall define “providing access to” in terms of having a list of URLs from which a human user can navigate to the full text of the resource. This means that both Unpaywall and BASE do not collect these full text resources, which is also why they do not face many of the challenges we described in the Introduction. Using this approach, we could say that the CORE Dataset provides access to approximately 139 million full texts, i.e. about 48% of our 291 million metadata records point to a URL from which a human can navigate to the full text. However, to people concerned with text and data mining of scientific literature, it makes little sense to count URLs pointing to many different domains on the Web as the number of full texts made available.

As a result, our 32.8 million statistic refers to the number of OA documents we identified, downloaded, extracted text from, validated their relationship to the metadata record and the full texts of which we host on the CORE servers and make available to others. In contrast, BASE and Unpaywall do not aggregate the full texts of the resources they provide access to and consequently do not offer the means to interact with the full texts of these resources or offer bulk download capability of these resources for text analytics over scholarly literature.

We have also integrated CORE data with the OpenMinTeD infrastructure, a European Commission funded project which aimed to provide a platform for text mining of scholarly literature in the cloud^6^.

### Use cases

A number of academia and industry partners have utilised CORE in their services. In this section we present three existing uses of CORE demonstrating how CORE can be utilised to support text and data mining use cases.

Since 2017, CORE has been collaborating with a range of scholarly search and discovery systems. These include Naver (https://naver.com/), Lean Library (https://www.leanlibrary.com/) and Ontochem (https://ontochem.com/). As part of this work, CORE serves as a provider of full text copies of reserch papers to existing records in these systems (Lean Library) or even supplies both metadata and full texts for indexing (Ontochem, NAVER). This collaboration also benefits CORE’s data providers as it expands and increases the visibility of their content.

In 2019, CORE entered into a collaboration with Turnitin, a global leader in plagiarism detection software. By using the CORE FastSync service, Turnitin’s proprietary web crawler searches through CORE’s global database of open access content and metadata to check for text similarity. This partnership enables Turnitin to significantly enlarge its content database in a fast and efficient manner. In turn, it also helps protect open access content from misuse, thus protecting authors and institutions.

As of February 2023, CORE Recommender^19^ is actively running in over 70 repositories including the University of Cambridge institutional repository and arXiv.org among others. The purpose of the recommender is to improve the discoverability of research outputs by providing suggestions for similar research papers both within the collection of the hosting repository and the CORE collection. Repository managers can install the recommender to advance the accessibility of other scientific papers and outreach to other scientific communities, since the CORE Recommender acts as a gate to millions of open access research papers. The recommender is integrated with the CORE search functionality and is also offered as a plugin for all repository software, for example EPrints, DSpace, etc. as well as open access journals and any other webpage. Based on the fact that CORE harvests open repositories, the recommender only displays research articles where the full text is available as open access, i.e. for immediate use, without access barriers or limited rights’ restrictions. Through the recommender, CORE promotes the widest discoverability and distribution of the open access scientific papers.

### Future work

An ongoing goal of CORE is to keep growing the collection to become a single point of access to all of world’s open access research. However, there are a number of other ways we are planning to improve both the size and ease of access to the collection. The CORE Harvesting System was designed to enable adding new harvesting steps and enrichment tasks. There remains scope for adding more of such enrichments. Some of these are machine learning powered, such as classification of scientific citations^20^. Further, CORE is currently developing new methodologies to identify and link different versions of the same article. The proposed system, titled CORE Works, will leverage CORE’s central position in the OA infrastructure landscape and will link different versions of the same paper using a unique identifier. We will continue to keep linking the CORE collection to scholarly entities from other services, thereby making CORE data participate in a global scholarly knowledge graph.

## Methods

In the Introduction section we focused on a a number of challenges researchers face when collecting research literature for text and data mining. In this section, we instead focus on the perspective of a research literature aggregator, i.e. a system whose goal is to continuously provide seamless access to research literature aggregated from thousands of data providers worldwide in a way that enables the resulting research publication collection to be used by others in production applications. We describe the challenges we had to overcome to build this collection and to keep it continuously up-to-date, and present the key technical innovations which allowed us to greatly increase the size of the CORE collection and become a leading provider of open access literature which we illustrate using our content growth statistics.

### CORE Harvesting system (CHARS)

CORE Harvesting System (CHARS) is the backbone of our harvesting process. CHARS uses the Harvesting Scheduler (Section CHARS_architecture) to select data providers to be processed next. It manages all the running processes (tasks) and ensures the available compute resources are well utilised.

Prior to implementing CHARS, CORE was centralised around data providers rather than around individual tasks needed to harvest and process these data providers (e.g. metadata download and parsing, full text download, etc.). Consequently, even though the scaling up and the continuation of this system was possible, the infrastructure was not horizontally scalable and the architecture suffered from tight coupling of services. This was not consistent with CORE’s high availability requirements and was regularly causing problems in the complexity of maintenance. In response to these challenges, we designed CHARS using a microservices architecture, i.e. using small manageable autonomous components that work together as part of a larger infrastructure^21^. One of the key benefits of microservices-oriented architecture is that the implementation focus can be put on the individual components which can be improved and redeployed as frequently as needed and independently of the rest of the infrastructure. As the process of open access content harvesting can be inherently split into individual consecutive tasks, a microservices-oriented architecture presents a natural fit for aggregation systems like CHARS.

#### Tasks involved in open access content harvesting

**Fig. 8:**
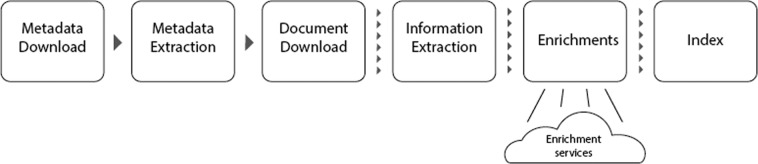
CORE Harvesting Pipeline. Each tasks’ output produces the input for the following task. In some cases the input is considered as a whole, for example all the content harvested from a data provider, while in other cases, the output is split in multiple small tasks performed on a record level.

The harvesting process can be described as a pipeline where each task performs a certain action and where the output of each task feeds into the next task. The input to this pipeline is a set of data providers and the final output is a system populated with records of research papers available from them. The main types of key tasks currently performed as part of CORE’s harvesting system are (Fig. 8):

1. **Metadata download:** The metadata exposed by a data provider via OAI-PMH are downloaded and stored in the file system (typically as an XML). The downloading process is sequential, i.e. a repository provides typically between 100–1,000 metadata records per request and a resumption token. This token is then used to provide the next batch. As a result, full harvesting can a significant amount of time (hours-days) for large data providers. Therefore, this process has been implemented to provide resilience to a range of communication failures.
2. **Metadata extraction**: Metadata extraction parses, cleans, and harmonises the downloaded metadata and stores them into the CORE internal data structure (database). The harmonisation and cleaning process addresses the fact that different data providers/repository platforms describe the same information in different ways (syntactic heterogeneity) as well as having different interpretations for the same information (semantic heterogeneity).
3. **Full text download**: Using links extracted from the metadata CORE attempts to download and store publication manuscripts. This process is non-trivial and is further described in the Using OAI-PMH for content harvesting section.
4. **Information extraction**: Plain text from the downloaded manuscripts is extracted and processed to create a semi-structured representation. This process includes a range of information extraction tasks, such as references extraction.
5. **Enrichment**: The enrichment task works by increasing both metadata and full text harvested from the data providers with additional data from multiple sources. Some of the enrichments are performed directly by specific tasks in the pipeline such as language detection and document type detection. The remaining enrichments that involve external datasets are performed externally and independently to the CHARS pipeline and ingested into the dataset as described in the Enrichments section.
6. **Indexing**: The final step in the harvesting pipeline is indexing the harvested data. The resulting index powers CORE’s services, including search, API and FastSync.

#### Scalable infrastructure requirements

Based on the experience obtained while developing and maintaining our harvesting system as well as taking into consideration the features of the CiteSeerX^22^ architecture, we have defined a set of requirements for a scalable harvesting infrastructure^8^. These requirements are generic and apply to any aggregation or digital library scenario. These requirements informed and are reflected in the architecture design of CHARS (Section CHARS architecture):

- **Easy to maintain:** The system should be easy to manage, maintain, fix, and improve.
- **High levels of automation:** The system should be completely autonomous while allowing manual interaction.
- **Fail fast:** Items in the harvesting pipeline should be validated immediately after a task is performed, instead of having only one and final validation at the end of the pipeline. This has the benefit of recognising issues and enabling fixes earlier in the process.
- **Easy to troubleshoot:** Possible code bugs should be easily discerned.
- **Distributed and scalable:** The addition of more compute resources should allow scalability, be transparent and replicable.
- **No single point of failure:** A single crash should not affect the whole harvesting pipeline, individual tasks should work independently.
- **Decoupled from user-facing systems:** Any failure in the ingestion processing services should not have an immediate impact on user-facing services.
- **Recoverable:** When a harvesting task stops, either manually or due to a failure, the system should be able to recover and resume the task without manual intervention.
- **Performance observable:** The system’s progress must be properly logged at all times and overlay monitoring services should be set up to provide a transparent overview of the services’ progress at all times, to allow early detection of scalability problems and identification of potential bottlenecks.

#### CHARS architecture

**Fig. 9:**
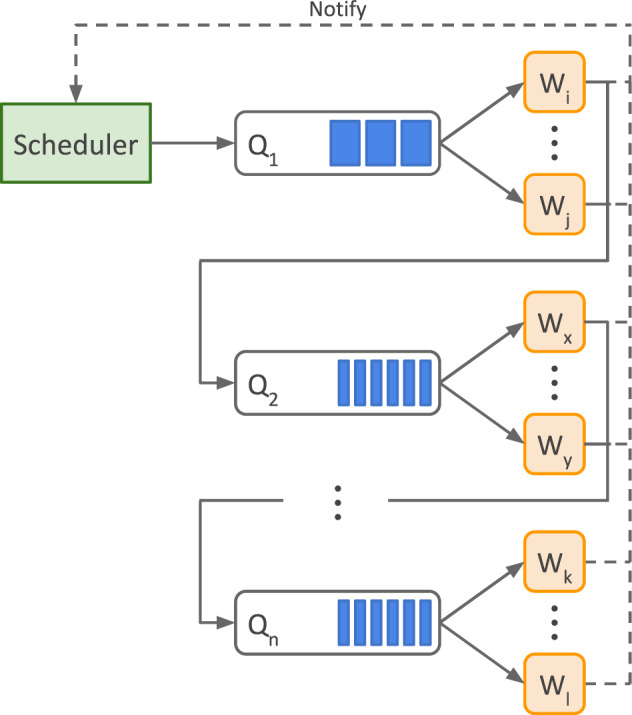
CORE Harvesting System.

An overview of CHARS is shown in Fig. 9. The system consists of the following main software components:

1. **Scheduler:** it becomes active when a task finishes. It monitors resource utilisation and selects and submits data providers to be harvested.
2. **Queue (Qn):** a messaging system that assists with communication between parts of the harvesting pipeline. Every individual task, such as metadata download, metadata parsing, full text download, and language detection, has its own message queue.
3. **Worker (W**_**i**_**):** an independent and standalone application capable of executing a specific task. Every individual task has its own set of workers.

A complete harvest of a data provider can be described as follows. When an existing task finishes, the scheduler is activated and informed of the result. It then uses the formula described in Appendix A to assign a score to each data provider. Depending on current resource utilisation, i.e. if there are any idle workers, and the number of data providers already scheduled for harvesting, the data provider with the highest score is then placed in the first queue Q_1_ which contains data providers scheduled for metadata download. Once one of the metadata download workers W_i_-W_j_ becomes available, a data provider is taken out of the queue and a new download of its metadata starts. Upon completion, the worker notifies the scheduler and, if the task is completed successfully, places the data provider in the next queue. This process continues until the data provider passes through the entire pipeline.

While some of the tasks in the pipeline need to be performed at the granularity of data providers, specifically metadata download and parsing, other tasks, such as full text extraction and language detection, can be performed at the granularity of individual records. While these tasks are originally scheduled at the granularity of data providers, only the individual records of a selected data provider which require processing are subsequently independently placed in the appropriate queue. Workers assigned to these tasks then process the individual records in the queue and they move through the pipeline once completed.

A more detailed description of CHARS, which includes technologies used to implement it, as well as other details can be found in^8^.

The harvesting scheduler is a component responsible for identifying data providers which need to be harvested next and placing these data providers in the harvesting queue. In the original design of CORE, our harvesting schedule was created manually, assigning the same harvesting frequency to every data provider. However, we found this approach inefficient as it does not scale due to the varying data providers size, differences in the update frequency of their databases and the maximum data delivery speeds of their repository platforms. To address these limitations, we designed the CHARS scheduler according to our new concept of “pro-active harvesting.” This means that the scheduler is event driven. It is triggered whenever the underlying hardware infrastructure has resources available to determine which data provider should be harvested next. The underlying idea is to maximise the number of ingested documents over a unit of time. The pseudocode and the formula we use to determine which repository to harvest next is described in Algorithm 1.

The size of the metadata download queue, i.e. the queue which represents an entry into the harvesting pipeline, is kept limited in order to keep the system responsive to the prioritisation of data providers. A long queue makes prioritising data providers harder, as it is not known beforehand how long the processing of a particular data provider will take. An appropriate size of the queue ensures a good balance between the reactivity and utilisation of the available resources.

#### Using OAI-PMH for content harvesting

We now describe the third key technical innovation which enables us to harvest full text content (as opposed to just metadata) from data providers using the OAI-PMH protocol. This process represents one step in the harvesting pipeline (Fig. 9), specifically, the third step which is activated after data provider metadata have been downloaded and parsed.

The OAI-PMH protocol was originally designed for metadata harvesting only, but due to its wide adoption and lack of alternatives it has been used as an entry point for full text harvesting from repositories. Full text harvesting is achieved by using URLs found in the metadata records to discover the location of the actual resource and subsequently downloading it^9^. We summarised the key challenges of this approach in the Challenges related to the use of OAI-PMH protocol for content harvesting section. The algorithm follows a depth first search strategy with prioritisation and finishes as soon as the first matching document is found.

The procedure works in the following way. First, all metadata records from a selected data provider with no full text are collected. Those records for which full text download was attempted within the retry period (*RP*) (usually six months) are filtered out. This is to avoid repeatedly downloading URLs that do not lead to the sought after documents. The downside of this approach is that if a data provider updates a link in the metadata, it might take up to the duration of the retry period to acquire the full text.

##### Algorithm 1

Formula for scheduling data providers for harvesting. This algorithm considers only a subset of the features available, new improvements in the algorithm might keep into account additional factors such as size of the provider, location, previous errors, etc.

Next, the records are further filtered using a set of rules and heuristics we developed to a) increase the chances of identifying the URL leading to the described document quickly and b) to ensure that we identify the correct document. These filtering rules include:

- **Accepted file extensions:** URLs are filtered according to a list of accepted file extensions. URLs ending with extensions such as*.pptx* that clearly indicate that the URL does not link to the required resource are removed from the list.
- **Same domain policy:** URLs in the OAI-PMH metadata can link to any resources and domains. For example, a common practice is to provide a link to the associated presentation, dataset, or another related resource. As these are often stored in external databases, filtering out all URLs that lead to an external domain, i.e. domain different than the domain of the data provider, presents a simple method of avoiding the download of resources which with very high likelihood do not represent the target document. Exceptions include *dx.doi.org* and *hdl.handle.net* domains whose purpose is to provide a persistent identifier pointing to the document. The same domain policy is disabled for data providers which are aggregators and link to many different domains by design.
- **Provider-specific crawling heuristics:** Many data providers follow a specific pattern when composing URLs. For example, a link to a full text document may be composed of the following parts: *data provider URL* + *record handle* + *.pdf*. For data providers utilising such patterns, URLs may be composed automatically where the relevant information (record handle) is known to us from the metadata. These generated URLs are then added to the list of URLs obtained from the metadata.
- **Prioritising certain URLs:** As it is more likely for PDF URL to contain the target record than for an HTML URL, the final step is to sort URLs according to file and URL type. Highest priority is assigned to URLs that uses repository software specific patterns to identify full text, document, and PDF filetypes, while the lowest priority is assigned to *hdl.handle.net* URLs.

The system then attempts to request the document at each URL and download it. After each download, checks are performed to determine whether the downloaded document represents the target record. Currently, the downloaded document has to be a valid PDF with a title matching the original metadata record. If the target record is identified, the downloaded document is stored and the download process for that record ends. If the downloaded document contains an HTML page, URLs are extracted from this page and filtered using the same method mentioned above. This is because it is common in some of the most widely used repository systems such as DSpace for the documents not to be directly referenced from within the metadata records. Instead, the metadata records typically link to an HTML overview page of the document. To deal with this problem, we use the concept of harvesting levels. A maximum harvesting level corresponds to the maximum search depth for the referenced document. The algorithm finishes either as soon as the first matching document is found or after all the available URLs up to the maximum harvesting level have been exhausted. Algorithm 2 describes our approach for collecting the full texts using the OAI-PMH protocol. The algorithm follows a depth first search strategy with prioritisation and finishes as soon as the first matching document is found.

##### Algorithm 2

Procedure for harvesting full text content using the OAI-PMH protocol.

#### CHARS limitations

Despite overcoming the key issues to scalable harvesting of content from repositories, there still remains a number of important challenges. The first relates to the difficulty of estimating the optimal number of workers in our system to run efficiently. While the worker allocation is still largely established empirically, we are investigating more sophisticated approaches based on formal models of distributed computation, such as Petri Nets. This will allow us to investigate new approaches to dynamically allocating and launching workers to optimise the usage of our resources.

#### Enrichments

**Fig. 10:**
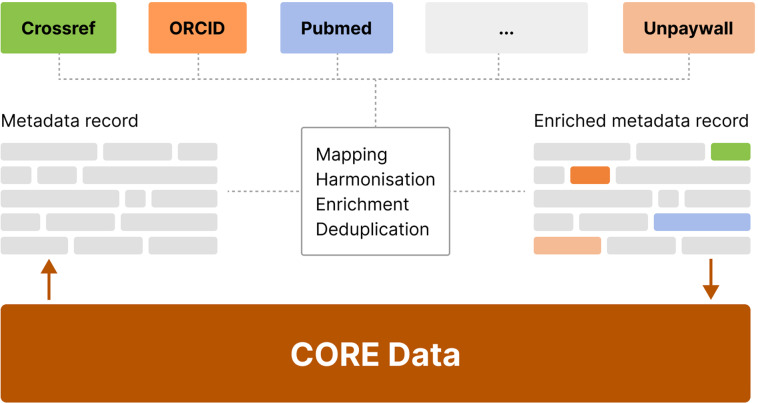
CORE Offline Enrichments.

Conceptually, two types of enrichment processes are used within CORE: 1) an *online enrichment process* enriching a single record at the time of it being processed by the CHARS pipeline and 2) a periodic *offline enrichment process* which enriches a record based on information in external datasets (Fig. 10).

#### Online enrichments

Online enrichments are fully integrated into the CHARS pipeline described earlier in this section. These enrichments generally involve the application of machine learning models and rule-based tools to gather additional insights about the record, such as language detection, document type detection. As opposed to offline enrichments, online enrichments are always performed just once for a given record. The following is a list of the current enrichments performed online:

- Article type detection: A machine learning algorithm assigns each publication one of the following four types: presentation, thesis, research paper, other. In the future we may include other types.
- Language identification: This task uses third-party libraries to identify the language based on the full text of a document. The resulting language is then compared to the one provided by the metadata record. Some heuristics are applied to disambiguate and harmonise languages.

#### Offline enrichments

Offline enrichments are carried out by means of gathering a range of information from large third-party scholarly datasets (research graphs). Such information includes metadata that do not necessarily change, such as a DOI identifier, as well as metadata that evolve, such as the number of citations. Especially due to the latter, CORE performs offline enrichments periodically, i.e. all records in CORE go through this process repeatedly at specified time intervals (currently once per month).

The process is depicted in Fig. 10. The initial mapping of a record is carried out using a DOI, if available. However, as the majority of records from repositories do not come with a DOI, we carry out a matching process against the Crossref database using a subset of metadata fields including title, authors and year. Once the mapping is performed, we can harmonise fields as well as gather a wide range of additional useful data from relevant external databases, thereby enriching the CORE record. Such data include, ORCID identifiers, citation information, additional links to freely available full texts, field of study information and PubMed identifiers. Our solution is based on a set of map-reduce tasks to enrich the dataset and implemented on a Cloudera Enterprise Data Hub (https://www.cloudera.com/products/enterprise-data-hub.html)^23–26^.

## Data Availability

CORE provides several large data dumps of the processed and aggregated data under the ODC-BY licence (https://core.ac.uk/documentation/dataset). The only condition for both commercial and non-commercial reuse of these datasets is to acknowledge the use of CORE in their outputs. Additionally, CORE makes its API and most recent data dump freely available to registered individual users and researchers. Please note that CORE claims no rights in the aggregated content itself which is open access and therefore freely available to everyone. All CORE data rights correspond to the sui generis database rights of the aggregated and processed collection. Licences for CORE services, such as the API and FastSync, are available for commercial users wishing to benefit from convenient access to CORE data with guaranteed level of customer support. The organisation running CORE, i.e. The Open University, is a charitable organisation fully committed to the Open Research mission. CORE is a signatory of the Principles of Open Scholarly Infrastructure (POSI) (https://openscholarlyinfrastructure.org/posse). No profit generation is practised. Instead, CORE’s income from licences to commercial parties is used solely to provide sustainability by means of enabling CORE to become less reliant on unstable project grants, thus offsetting and reducing the cost of CORE to the taxpayer. This is done in full compliance with the principles and best practices of sustainable open science infrastructure.

## Code availability

CORE consists of multiple services. Most of our source code is open source and available in our public repository on GitHub (https://github.com/oacore/). As of today, we are unfortunately not yet able to provide the source code to our data ingestion module. However, as we want to be as transparent as possible with our community, we have documented in this paper the key algorithms and processes which we apply using pseudocode.
